# Seroprevalence of nasal myiasis in camels determined by indirect enzyme-linked immunosorbent assay utilizing the most diagnostic *Cephalopina titillator* larval antigens

**DOI:** 10.14202/vetworld.2022.2830-2835

**Published:** 2022-12-13

**Authors:** Noha M. F. Hassan, Doaa Sedky, Nadia M. T. Abu El Ezz, Eman E. El Shanawany

**Affiliations:** Department of Parasitology and Animal Diseases, National Research Centre, P.O. Box 12622, 133 El-Behouth Street, Dokki, Cairo, Egypt

**Keywords:** camel, *Cephalopina titillator*, diagnosis, enzyme-linked immunosorbent assay, larval antigens, nasal myiasis, seroprevalence

## Abstract

**Background and Aim:**

Nasal myiasis is a serious parasitic disease among camels caused by *Cephalopina titillator* larvae that negatively affect animal health and production globally. The diagnosis of the infestation relies on postmortem examination of the head region, which considers a cause impeding treatment of live animals and may be misdiagnosed as central nervous system disorders. This study aimed to identify the most diagnostic larval antigen with the capacity for monitoring *C. titillator* infestation, and to estimate the seroprevalence of nasal myiasis in camels in Egypt, using indirect enzyme-linked immunosorbent assay (ELISA).

**Materials and Methods:**

Three hundred and six male camels of Egyptian and Sudanese breeds, aged 2–5 years, were clinically evaluated for respiratory and/or nervous disorders in Cairo Governorate, Egypt. At the time of slaughter, blood samples were collected from all examined animals. The postmortem examination of 38 animals was conducted. Salivary glands, hemolymph, and somatic antigens were extracted from the second and third larval instars.

**Results:**

The results revealed that the salivary gland antigen was the most potent antigen in detecting *C. titillator* specific total IgG antibodies compared to haemolymph and crude somatic antigens. Using receiver-operating characteristic curves and area under the curve, the salivary gland antigen had a sensitivity of 91.67% and a specificity of 92.31%, respectively. It has the highest positive predictive value, 95.7%, and negative predictive value, 85.7%. However, using somatic and hemolymph antigens revealed a sensitivity of 79.17% and 70.83% and a specificity of 76.9% and 84.6%, respectively. There was complete concordance between ELISA results and autopsy findings (true positive). One hundred and forty out of 306 (45.8%) camel serum samples were found to contain *C. titillator*.

**Conclusion:**

This study demonstrated that salivary gland antigen is more effective than somatic and hemolymph antigens in accurately detecting nasal myiasis in camels. In addition, determining the seroprevalence of nasal myiasis with the salivary gland antigen through indirect ELISA revealed that it is a prevalent disease among camels in Egypt. Periodic surveillance of the *C. titillator* prevalence is necessary for effective management and control measures.

## Introduction

The camel, *Camelus dromedarius* L. 1758, is an essential vital multipurpose domestic animal in arid and semi-arid regions worldwide, serving as the primary source of meat, milk, and hides [1, 2]. Parasitic diseases are a serious issue that negatively impacts camels’ productivity. In addition to external parasites such as ticks, scabies, and ringworms, camels suffer from internal parasites such as *Strongyles* spp., *Eimeria* spp., and hydatid cysts [3, 4]. Among the most urgent parasitic diseases, nasal myiasis is widespread in camels, causing severe economic losses and endangering animal health, production, and welfare [5–8]. The disease is caused by the nasal bot fly larvae of *Cephalopina titillator* (Family Oestridae: Diptera). It is an obligate parasite of camels in which the female ostrid fly deposits the first larval instars directly at the nostrils of camels, after which they crawl up to the nasopharynx and paranasal sinuses and remain attached to the mucous membrane for approximately 11 months, during which they molt twice [9, 10], causing extensive irritation and mucosal damage. The infested camels experience excessive nasal discharge, head shaking, loss of appetite, decreased milk production, abortion, and respiratory disorders [11]. Secondary bacterial invasion may cause pyogenic infection and pneumonia [12].

Furthermore, the larvae may reach the cranial cavity, causing meningitis, and infested camels may exhibit symptoms similar to cranial coeneurosis. The upward movement of the larva could cause mechanical damage and bone penetration of the ethmoid. Meningitis and secondary bacterial infection of the cerebrospinal canal may have caused the infected animal’s demise [13, 14].

The long attachment period of larval instars to the nasopharyngeal mucosa can trigger an immune response in the host. Larvae stimulate cellular and humeral immune responses; antigens stimulate natural killer cells, which produce interferon γ to enhance T helper cells, which in turn activate B cells that release immunoglobulins [15].

Numerous postmortem examination-based studies have reported the global prevalence of camel nasal myiasis [16–18]. The development of a reliable serological diagnostic test is greatly beneficial for rapid intervention with appropriate treatment, especially in detecting infection in live animals and distinguishing it from central nervous system disorders [19, 20].

We sought to assess *C. titillator* sensitivity in crude extracts of larval antigens, including digestive tubule, excretory-secretory products, and salivary glands, for detecting anti-*C. titillator* antibodies in camel sera [21] and in mucus samples [22] through indirect enzyme-linked immunosorbent assay (ELISA).

Therefore, this study aims to determine the most diagnostic *C. titillator* larval antigen for accurate diagnosis and indirect ELISA monitoring of nasal myiasis prevalence in camels in Egypt.

## Materials and Methods

### Ethical approval

Ethical permission for the use of animals was approved by institutional guidelines of the National Research Centre’s Animal Research Committee (Approval number 1472032022). All procedures involving animals were performed solely by licensed personnel.

### Study period and location

The study was conducted from December 2021 to February 2022 at the Cairo Governorate 30°02′40″N 31°14′09″E in Egypt. The samples were processed at the Parasitology and Animal Diseases Department, Veterinary Research Institute, National Research Centre, Egypt.

### Animals and sampling

A total of 366 *male* camels of Egyptian and Sudanese breeds between the ages of 2 and 5 were utilized. Before slaughtering, the animals were clinically examined for respiratory and nervous manifestations. At the time of slaughter, 306 blood samples were collected from the camels. To assign the parasitologically positive and negative animals’ sera, 38 blood samples out of 306 were collected from camels whose heads and nasopharyngeal regions had been subjected to postmortem examination (gold standard). Separated serum samples were stored at −20°C until use.

### Larvae

**Figure-1:**
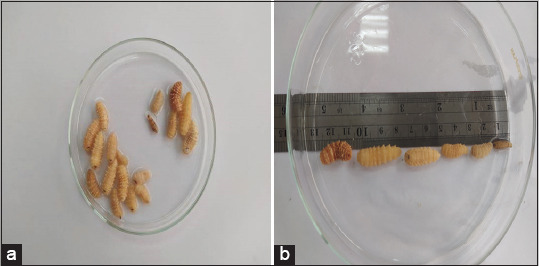
(a) The larvae of *Cephalopina titillator* collected from infested camels. (b) Different larval stages; first, second, third stage, and pupa of *C. titillator*.

Various larval stages of *C. titillator* were extracted from the turbinate of slaughtered camels (Figures-1a and b). The larvae were identified using the morphological key adopted by Zumpt [9]. Collected larvae were washed with distilled water, followed by phosphate buffer saline with a pH of 7.2.

### Antigens

Three different antigens were prepared from *C. titillator* larvae, according to Tabouret *et al*. [23]. The antigen of salivary glands was obtained by extracting salivary glands from collected larval instars using a dissecting microscope. Whole larval instars were homogenized in phosphate-buffered saline (PBS) with a pH of 7.2 to produce crude somatic antigens. The hemolymph antigen was collected from various larval instars. Each antigen was ground and sonicated in cold PBS before being centrifuged for 20 min at 250× g in a cooling centrifuge. The supernatant was then collected, aliquoted, and stored at *−*20°C until use. According to a previous study [24], the total protein concentrations of various antigens were estimated.

### Diagnostic potency of *C. titillator* larvae antigens

The immunological interactions of the somatic, salivary gland, and heamolymph antigens prepared with the infested and healthy camel sera were determined by indirect ELISA as described by Abdel-Rahman *et al*. [25]. Subsequently, the diagnostic efficacy of prepared antigens was evaluated using two-fold serial dilutions of positive infected sera. Total immunoglobulin G (IgG) antibodies specific to the *C. titillator* were detected in camel serum samples using the most potent antigen evaluated. Next, the optimal antigens’ concentrations were determined using a preliminary checkerboard titration test.

Briefly, the plate was coated with three prepared antigens, including somatic, salivary gland, and hemolymph, in coating buffer (100 μL/well) and then incubated at 4°C overnight. The unbinding sites were blocked with 0.1 M bovine serum albumin in the coating buffer. The plate was incubated for 1 h at room temperature (27°C). The plate was repeatedly washed, and serum samples (100 μL/well) were diluted (1:100) and incubated at 37°C for 90 min. In the case of two-fold serial dilution, serum was not diluted in the first well. After washing, the conjugate was incubated for 1 h at 37°C with 100 µl of protein A (Sigma Chem. Co., St. Louis, USA). Then, 100 μL/well of the substrate (Orthophenylenediamine) was added, and the plate was read spectrophotometrically at 450 nm. The cutoff point of optical density (OD) values was determined as described by El Shanawany *et al*. [26, 27].

### Statistical analysis

The diagnostic accuracy of the various antigens derived from *C. titillator* larvae was evaluated using the receiver-operating characteristic (ROC) area under the curve (AUC). The AUC values were evaluated as follows: AUC< 0.5 was deemed noninformative; 0.5 < AUC < 0.7 indicated low accuracy; 0.7 < AUC < 0.9, moderate accuracy; and 0.9 < AUC < 1 high accuracy [28]. The diagnostic accuracy was determined by calculating the sensitivity, specificity, and efficiency, and the values are expressed as a percentage. GraphPad Prism version 6.0 (GraphPad Software, La Jolla, CA, USA) MedCalc, a statistical software, was used to analyze and plot all statistics.

## Results

### Clinical and postmortem inspection

Before slaughter, a clinical examination of live camels revealed restlessness, nasal discharge, nasal mucosal congestion, snorting while breathing, sneezing, dropping larvae to the ground, a high respiratory rate, and neurological symptoms.

A postmortem examination of 38 camels revealed that 63.15% (24/38) of the examined camels were infected with *C. titillator* instars. Most larvae were found in the nasopharyngeal and frontal sinuses, as well as some turbinates and ethmoid regions. In the presence of the larvae, the mucous membranes were congested and contained thick bloody mucus.

### Evaluation of larval antigens in the detection of *C. titillator*-specific IgG in infested camel sera

**Figure-2:**
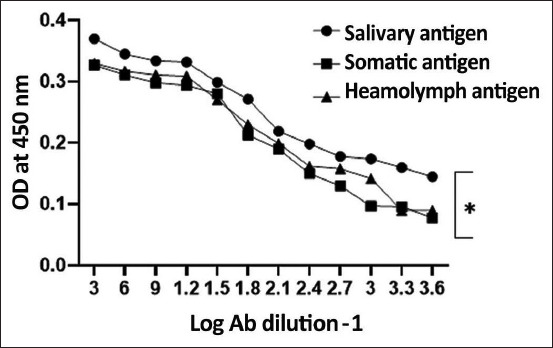
Comparative diagnostic potential of the salivary gland, somatic, and hemolymph antigens of *Cephalopina titillator* larva.

The diagnostic efficacy of prepared larval antigens (somatic, salivary gland, and hemolymph) was evaluated by analyzing the reactivity of these antigens with camel sera serially diluted two-fold. The comparison of optical densities revealed a statistically significant difference between the three prepared antigens. The salivary gland antigen was statistically (p < 0.05) the most effective antigen for detecting *C. titillator*-specific total IgG antibodies as measured by indirect ELISA (Figure-2).

### Sensitivity and specificity of prepared antigens from larvae

**Figure-3:**
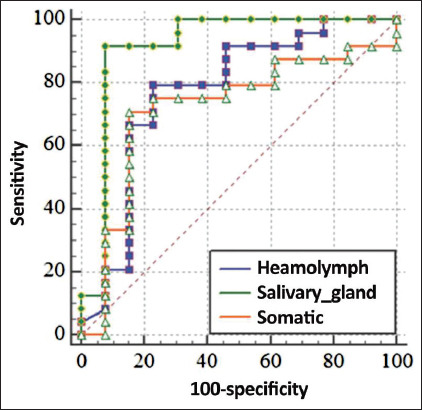
Graphical representation of receiver-operating characteristic curve analysis to evaluate the diagnostic potency of three prepared antigens from *Cephalopina titillator* larva used for diagnosis of camel nasal myiasis.

To assess the sensitivity and specificity of the three antigens for the diagnosis of camel nasal myiasis, camel nasal myiasis ROC curves and AUC were generated (Figure-3). Considering the serological results, the ELISA with salivary gland antigen showed the best discrimination, with a sensitivity of 91.67% and a specificity of 92.31%, as well as a positive predictive value of 95.7% and a negative predictive value of 85.7%. In contrast, the ELISAs with somatic and hemolymph antigens showed sensitivity of 79.17% and 70.83%, respectively and specificity of 76.9% and 84.6%, respectively, and the specificity of the range of possible AUC values is between 0.7 and 1.0 (moderate diagnostic ability) to 0.9–1.0 (perfect diagnostic ability). The salivary gland antigen revealed the greatest value (AUC = 0.923), while the somatic antigen revealed the least (AUC = 0.718) (Table-1). In analyses conducted, the p-value was <0.0001, indicating that salivary gland antigen can distinguish between infested and non-infested animals.

**Table-1:** Efficacy parameters of *C. titillator* somatic, salivary gland, and hemolymph antigens in the diagnosis of camel nasal myiasis using direct ELISA

Antigen	Sensitivity %	Specificity %	PPV %	NPV%	Diagnostic accuracy (%)	AUC
Salivary gland	91.67	92.31	95.7	85.7	92.02	0.923
Somatic	79.17	76.9	86.4	66.7	77.94	0.718
Hemolymph	70.83	84.6	89.5	61.1	78.3	0.779

PPV=Positive predictive value, NPV=Negative predictive value, AUC=Area under the curve, *C. titillator*=*Cephalopina titillator*, ELISA=Enzyme-linked immunosorbent assay

### Seroprevalence of camel nasal myiasis by ELISA

**Figure-4:**
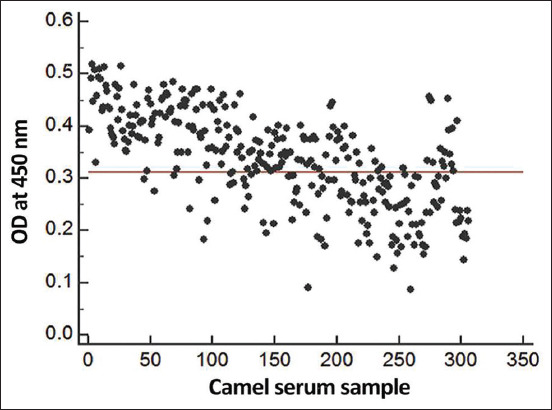
Camel nasal myiasis seroprevalence by indirect enzyme-linked immunosorbent assay in Egypt. The cutoff absorbance for seropositivity was 0.312.

The figure depicts the distribution of IgG antibodies in serum samples as determined by ELISA (Figure-4). There was perfect concordance between ELISA results and autopsy findings (gold-standard test). From a total of 306 tested samples of camel serum, 140 were seropositive, for a seropositivity rate of 45.8% by IgG ELISA. The cutoff OD value for the standard ELISA under consideration was 0.312.

## Discussion

Nasal myiasis is a severe disease caused by the obligate parasite *C. titillator* that negatively impacts the reproduction and production of camels. The clinical examination of live, infested camels revealed a variety of disease symptoms, including the previously observed respiratory impairment [29–31]. A few investigated cases exhibited nervous symptoms that could be misdiagnosed as neurological disorders, for example, “Shimbir,” rabies, and *Coenurus cerebralis* [32]. Necropsy examination is required to monitor the prevalence of nasal myiasis in camels and to distinguish it from other diseases. The postmortem examination of the studied camels revealed a large number of attached and impeded larvae in the mucous membrane of the nasopharyngeal and frontal sinuses, which is believed to be the primary cause of various inflammatory and degenerative changes, as well as obstruction of the air passage. It was reported that *C. titillator* is a larva of the common nasal botfly that infests camel populations in African and Asian countries [33]. Moreover, Spratt [34] also recorded the larval oestrid *C. titillator* for the first time as a parasite of camels in Australia. The current study revealed that 63.15% of camels examined after slaughter were infected with *C. titillator*. This prevalence may be comparable to that observed in China [35] where it was 54.2%, the eastern region of Saudi Arabia [36] where it was 41%, Iran [13] where it was 58.1%, and Iran [14]; where it was 71.4%. In contrast, a high prevalence of *C. titillato*r was 100% in western Sudan [12]. Egypt’s North Sinai Governorate [37] reported a lower incidence rate (25%), while Iraq’s [31] incidence rate was 40.07%. These variations may be attributable to differences in geographical regions, seasonal climatic conditions, and the immune status of animals [38, 39].

The diagnosis of nasal myiasis is primarily determined by postmortem examination. In addition to providing valuable opportunities for the treatment of living animals, accurate detection of the infestation prevents misdiagnosis of central nervous system disorders. To date, no special diagnostic kit has been discovered to be utilized for monitoring antibodies against *C. titillator*. Historically, various antigens have been used to detect *C. titillator* infestation with variable efficacy based on the protein content of the prepared antigen and the corresponding immune responses. Therefore, this study aimed to identify the most potent and diagnostic *C. titillator* larval antigen capable of detecting specific total IgG antibodies via indirect ELISA [20, 22, 40]. It was evident that the salivary gland antigen was the most powerful compared to hemolymph and somatic antigens. In experimentally infected sheep with nasal bot fly, salivary gland antigen was shown [41] to elicit a strong humeral response against *Oestrus ovis*. In addition, Attia *et al*. [40] described the immunogenic efficacy of salivary gland antigens in inducing an antibody response against *C. titillator* infestation. In addition, researchers validated camels’ [20] high diagnostic value of the organ’s content antigen (including salivary glands and digestive system) in detecting nasal myiasis. This could be the result of salivary glands secreting the contents of different instars through the mucus membrane of the host, which remained attached for an extended period and induced a high IgG response [10, 23]. In this study, the diagnostic accuracy of the hemolymph antigen was higher (78.3%) than that of the crude somatic antigen (77.8%). Functionally, the hemolymph resembles the blood and lymph of vertebrates [42]. Many insects’ immune responses occur as hemocytes pass passively with the hemolymph [43]. Concerning the development of an immune response against the prepared crude somatic antigen, the antigen may be returned to the cuticle protein during or after the molting of instars [44].

Most previous studies have relied on the PM examination as the primary method for determining the prevalence of the infestation. Enzyme-linked immunosorbent assay is regarded as a simple, inexpensive, and accurate immunological diagnostic tool capable of reducing the incidence of morbidity through early disease diagnosis [40]. Periodic surveillance of *C. titillator* prevalence among camels is required to effectively manage and control nasal myiasis. The seroprevalence of *C. titillator* by ELISA using the salivary gland antigen as the most immunogenic antigen, was 45% of the examined camels. Using indirect ELISA, the seroprevalence was found to be 90% [40] and 83.33% [20], respectively. These variations may be attributable to the immunological status of animals, sex, age, and seasonal and climatic variations in various Egyptian regions. By detecting antisalivary gland antigen antibodies [23], they discovered that 60% and 42% of sheep were infected with nasal botflies during the summer and winter seasons, respectively.

## Conclusion

Nasal myiasis is a prevalent disease among camel breeds found in Egypt. The salivary gland antigen is superior in eliciting an antibody response against *C. titillator* infestation compared to crude somatic antigens and hemolymph. Periodic monitoring of the prevalence of *C. titillator* in camels is required to manage and control nasal myiasis effectively.

## Authors’ Contributions

NMFH and EEE: Designed the study. NMFH, DS, and NMTA: Collected the samples. NMFH, DS, and EEE: Antigen preparation. NMFH and EEE: Conducted the seroprevalence of the study. NMFH and EEE: Collected the data and edited the manuscript. EEE and NMFH: Statistical analysis. NMFH, DS, NMTA, and EEE: Reviewed the manuscript. All authors have read and approved the final manuscript.
